# Modulating TFEB lysosomal function for aging and Alzheimer's disease

**DOI:** 10.1002/alz70861_108380

**Published:** 2025-12-23

**Authors:** Marco Sardiello

**Affiliations:** ^1^ Washington University in St. Louis, St. Louis, MO USA

## Abstract

**Background:**

Transcription factor EB (TFEB) is a master regulator of lysosomal biogenesis and autophagy that plays a key role in the regulation of cellular clearance pathways. TFEB is regulated via a complex array of post‐translational modifications (PTMs) that limit or enhance its activity.

**Method:**

We generated and analyzed a transgenic mouse line with neuronal expression of a TFEB transgene modified to resist inhibitory regulation. We also used a combination of kinome screening, biochemical assays, and protein modification to identify proteins relevant in TFEB turnover and function.

**Result:**

TFEB mice have a significantly elongated lifespan and improved metabolism, immune function, and liver health, indicating a parallel extension of healthspan. We identify IKK (inhibitor of κB kinase) as a TFEB direct modifier. IKK‐mediated phosphorylation of TFEB triggers TFEB ubiquitination by the E3 ligase β‐TrCP2 (β‐Transducin repeat‐containing protein 2), which tags TFEB for proteasomal degradation. Modified TFEB constructs that abolish phosphorylation by IKK or ubiquitination by β‐TrCP2 show much increased stability, while remaining sensitive to autophagy‐ or stress‐related stimuli. These stabilized constructs drive increased expression of TFEB target genes and promote clearance of tau aggregates in a model of Alzheimer’s disease.

**Conclusion:**

These findings position TFEB as a promising target for interventions in aging and neurodegeneration and highlight the IKK‐TFEB axis as a therapeutic entry point in Alzheimer’s pathology.

